# Developments in Non-Intercalating Bacterial Topoisomerase Inhibitors: Allosteric and ATPase Inhibitors of DNA Gyrase and Topoisomerase IV

**DOI:** 10.3390/ph16020261

**Published:** 2023-02-08

**Authors:** Scott Grossman, Colin W. G. Fishwick, Martin J. McPhillie

**Affiliations:** School of Chemistry, University of Leeds, Leeds LS2 9JT, UK

**Keywords:** topoisomerase, DNA gyrase, topo IV, antibiotic, allosteric, ATPase

## Abstract

Increases in antibiotic usage and antimicrobial resistance occurrence have caused a dramatic reduction in the effectiveness of many frontline antimicrobial treatments. Topoisomerase inhibitors including fluoroquinolones are broad-spectrum antibiotics used to treat a range of infections, which stabilise a topoisomerase-DNA cleavage complex via intercalation of the bound DNA. However, these are subject to bacterial resistance, predominantly in the form of single-nucleotide polymorphisms in the active site. Significant research has been undertaken searching for novel bioactive molecules capable of inhibiting bacterial topoisomerases at sites distal to the fluoroquinolone binding site. Notably, researchers have undertaken searches for anti-infective agents that can inhibit topoisomerases through alternate mechanisms. This review summarises work looking at the inhibition of topoisomerases predominantly through non-intercalating agents, including those acting at a novel allosteric site, ATPase domain inhibitors, and those offering unique binding modes and mechanisms of action.

## 1. Introduction

Infectious disease contributes significantly to worldwide mortality rates, with over 10 million deaths attributed to this class of disease in 2019 [1]. Following the publication of the O’Neill report in 2016, it was shown that rising trends in antimicrobial resistance (AMR) will have profound effects on both mortality rates as well as the global economy [2]. Data from the GRAM report suggested that in 2019, AMR infections were a factor in nearly 5 million deaths, directly causing mortality in 1.2 million cases [3]. As such, there is an urgent need to curb deaths due to infectious diseases, particularly through the development of novel antibiotics.

Resistant infections are increasing due to a multitude of reasons, including overprescribing antibiotics, their use in agriculture, and poor public understanding of the causes and implications of antibiotic resistance [4,5,6]. Current estimates predict that should these trends remain on course, by 2050, deaths due to AMR could exceed 10 million, more than currently die worldwide from all cancers combined [2]. Therefore, one approach to reducing AMR is the production of new treatments, particularly drugs with new mechanisms of action, in order to evade pre-existing resistance methods utilised by bacteria.

Broad-spectrum antibiotic activity can be achieved through the inhibition of essential proteins and enzymes found to be relatively well conserved across bacteria. For example, topoisomerases are found in all prokaryotes and eukaryotes, and some viruses [7]. Topoisomerases are responsible for topological changes in DNA, notably changes in supercoiling, decatenation, and unknotting, and are heavily involved in the replication and transcription processes by regulating supercoiling and by decatenating intertwined DNA strands [8]. Without these topological adjustments, DNA would remain unusable, leading to cell death.

Topoisomerases are divided into classes I and II dependent on whether they act via a single-strand DNA break (type I) or a double-strand DNA break (type II), with bacteria generally containing up to four examples [9]. For example, *Escherichia coli* contains topoisomerase I and topoisomerase III (type I), both of which are dimeric and reduce negative supercoiling in DNA [10]. Within type II, *E. coli* produces topoisomerase II (DNA gyrase) and topoisomerase IV (topo IV). DNA gyrase is found to introduce negative supercoils into DNA, whilst topo IV decatenates DNA and relaxes positive supercoiling. Whilst type I topoisomerases are, in theory, suitable drug targets, to date, the only clinically used drugs targeting bacterial topoisomerases are type II inhibitors.

**Figure 1:**
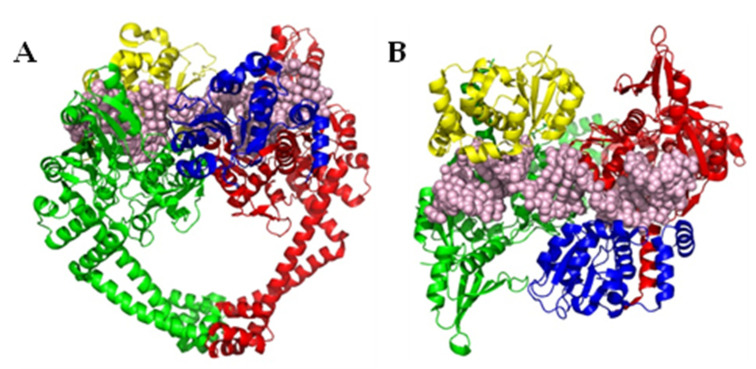
(**A**) Front view of *Staphylococcus aureus* DNA gyrase (PDB ID 6QX2 [13]), representative of the structure of type II topoisomerases. (**B**) Top view of *S. aureus* DNA gyrase, rotated 90° forwards from (**A**). The GyrA/ParC units of topoisomerase (green and red cartoons) are known to be critical to the actions of DNA strand breakage and covalent linkage of the phosphate backbone (whole DNA segment in pink spheres) to a conserved tyrosine residue in the active site, whilst the GyrB/ParE units (yellow and blue cartoons) are required for interactions with ATP. Both of these interactions are putative targets for topoisomerase inhibitors.

**Figure 2:**
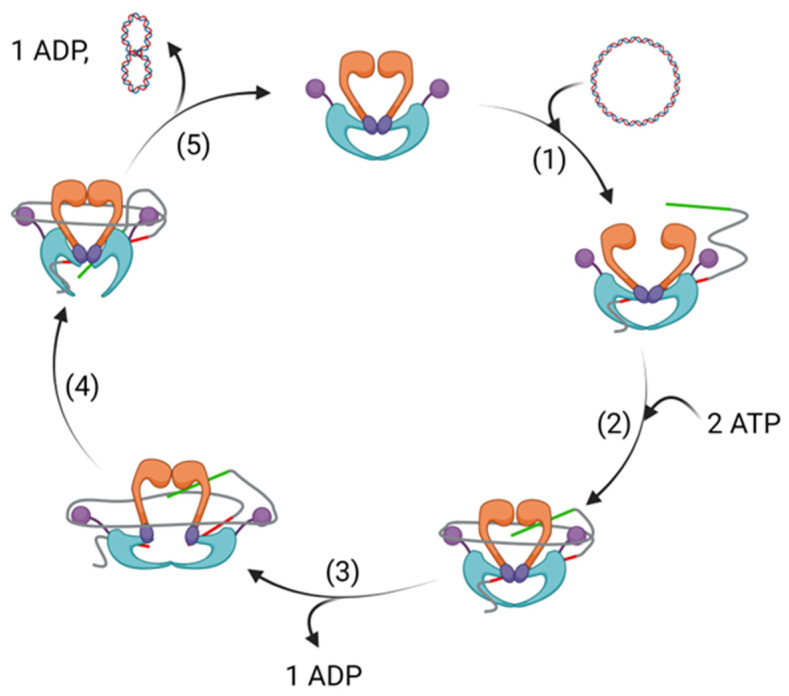
A generalised catalytic cycle of a topoisomerase. This figure depicts a cycle increasing supercoiling in circular DNA, though a similar cycle is used to reduce supercoiling, and decatenate and unknot DNA; GyrA units in blue; GyrB units in orange; G segment of DNA in red; T segment of DNA in green; non-interacting DNA in grey. (1) DNA interacts with the topoisomerase, and the G segment associates at the interface between the GyrA and GyrB subunits. (2) The non-interacting DNA loops around the topoisomerase and the T segment enters the topoisomerase, positioning itself above the G segment. The binding of ATP to the ATPase domain of GyrB causes the N-terminal gate to shut. (3) The G segment is cleaved, creating a change in conformation, enabling strand passage. (4) The T segment passes through the cleavage complex, altering the link number by two, and leaves via an exit gate. (5) Religation of the G segment, promoted by ATP hydrolysis, reopens the N-terminal gate, through which it leaves in its supercoiled form. The release of ADP reverts the conformation of topoisomerase back to its apo form.

DNA gyrase and topo IV are tetrameric proteins formed from two copies each of two proteins. DNA gyrase consists of two subunits of GyrA and two subunits of GyrB, with the protein found in the form A_2_B_2_ (Figure 1). Likewise, with topo IV, two subunits each of ParC and ParE form a homologous A_2_B_2_ tetramer [11]. In DNA gyrase, the enzyme functions through a complex mechanism, detailed studies of which have been described elsewhere [12]. Briefly, a segment of DNA (G segment) associates with DNA gyrase at the interface of the GyrA and GyrB subunits, and is wrapped around the enzyme (Figure 2). Upon the winding of approximately 130 bp around the enzyme, the conformation allows for a different segment of the same DNA strand (T segment) to enter the complex through the N-terminal gate of the GyrB subunits, positioning it above the G segment of DNA already associated.

The GyrB subunit contains an ATPase domain, and the binding of ATP to this protein causes the N gate to shut, trapping the T segment, followed by double strand cleavage of the G segment and subsequent covalent linkage of the phosphate backbone to a conserved tyrosine located on the GyrA subunit [14]. The strand break allows the T segment to pass through the broken G segment, thereby changing the link number by two, and performing its function to decatenate or alter supercoiling. The T segment leaves the protein through an exit gate, formed through a conformational change of the GyrA subunits. Religation of the broken strand, promoted through ATP hydrolysis, reforms the G segment, which exits via the N gate, reopened through the conversion of ATP to ADP and its subsequent dissociation from the ATPase domain. Topo IV acts through a similar mechanism, though with homologous subunits ParC and ParE forming the complex with DNA in place of GyrA and GyrB, respectively. Whilst ATP provides the energy required for DNA gyrase to introduce negative supercoils into DNA, in the absence of ATP, it relaxes negative supercoiling in DNA.

**Figure 3:**
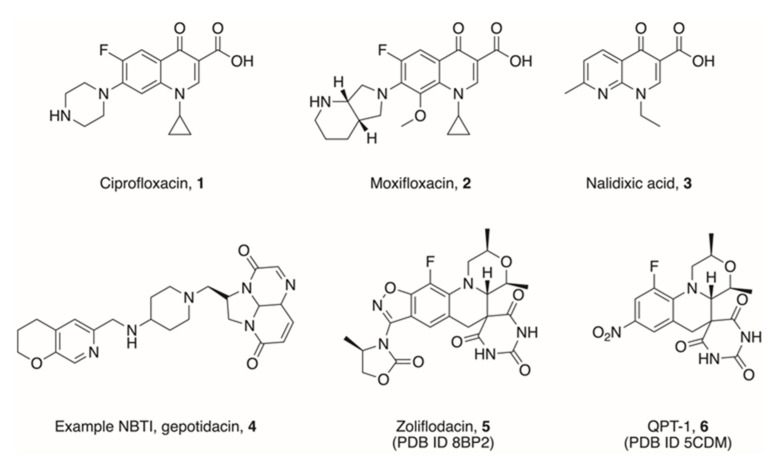
Examples of DNA intercalating drugs. Ciprofloxacin **1** and moxifloxacin **2** are examples of fluoroquinolones (FQ), which are readily used as frontline drugs currently. They are both derivatives of an earlier class of drug, 1,8-naphthyridinones, of which nalidixic acid **3** was the first-in-class. Gepotidacin **4** is an example of a class of topoisomerase inhibitor, which intercalates DNA at an alternative site, leading to a different mechanism of action. Zoliflodacin **5** and QPT-1 **6** are examples of spiropyrimidinetriones, found to overlap the FQ site, but act with an alternate mode of inhibition.

The crucial interaction between the GyrA/ParC unit and DNA is the target for the fluoroquinolone (FQ) class of drugs [15]. Following DNA strand breakage, the FQs are able to stabilise the cleavage complex by stacking between DNA base pairs at the site of cleavage, thus preventing further advancement of the strand passage and religation process. The FQs are often referred to as being ‘gyrase poisons’. Many FQs are used as frontline drugs, such as ciprofloxacin and moxifloxacin (**1** and **2**, Figure 3)**,** in part due to their broad spectrum of activity across both Gram-positive and Gram-negative bacteria [16].

FQs have been in use since the 1970s, though the precursor drug nalidixic acid (**3**, Figure 3), a 1,8-naphthyridinone, was discovered in 1962 [17]. Since then, they have been increasingly used as frontline drugs to treat a wide range of infections, particularly urinary tract infections [18]. They do, however, have several adverse side-effects, which can affect patients significantly. Of note, treatment with FQs has been shown to increase the risk of tendon disorders, including tendon ruptures and tendinitis [19]. The severity of these risks led the FDA to introduce a black box warning on many FQ antibiotics [20]. Moreover, FQ use has been shown to increase the bacterial SOS response, causing increased mutation and antibiotic resistance in persister cells [21,22].

Although usage in high-income countries has stabilised since the year 2000, overall usage is increasing steadily worldwide, with findings by Browne et al. suggesting that global use per capita has approximately doubled since 2000 [23]. With this trend, an increase in FQ resistance has emerged in pathogens affecting humans, wildlife, farmed animals, and aquatic environments, with multiple mutations frequently observed in clinical isolates, notably single-nucleotide polymorphisms (SNP) at sites GyrA^83^, GyrA^87^, ParC^80^, and ParC^84^ (*E. coli* numbering).

Whilst plasmid-mediated quinolone resistance does occur, SNP occurrence is the most common form of FQ resistance. Hamed et al. found that, in their study of 169 cancer patients suffering infection due to ciprofloxacin-resistant pathogens, all isolates contained at least one mutation in GyrA, and 93.3% had a further mutation in ParC, thereby inhibiting the crucial interaction between ciprofloxacin and the GyrA/ParC subunit [24]. Variations in the FQ core have subsequently been developed, which evade these forms of resistance, including using a 2-pyridone, quinazolinedione, isothiazoloquinolone, and oxazoloquinolin-2-one core [25,26,27,28].

As FQ resistance poses a significant threat to global healthcare, drugs targeting topoisomerases that further differentiate from FQs through distinct binding positions and mechanisms of inhibition are highly desirable. One area where researchers have made progress in is the development of Novel Bacterial Topoisomerase Inhibitors (NBTIs), such as gepotidacin (**4**, Figure 3). These compounds, similarly to FQs, act as DNA intercalating agents, though they occupy a different site within the cleavage complex to evade some FQ resistance mechanisms. Successes in NBTI developments have been thoroughly reviewed elsewhere [29,30,31]. 

One further interesting scaffold which has been developed in recent years is that of the spiropyrimidinetrione, the most studied example of which is zoliflodacin, also known as AZD0914 and ETX0914 (**5**, Figure 3) [32,33]. Initially, this compound was used to target *Neisseria gonorrhoeae*, and is currently in Phase III trials (NCT number NCT03959527), but it shows further strong activity against a range of other bacteria [34,35]. Work by Basarab et al. showed that zoliflodacin displays a novel mode of inhibition with respect to ciprofloxacin, and interacts with GyrB more strongly than GyrA [32,36], whilst still intercalating DNA at a site overlapping that of FQs, as evidenced by crystallographic data on both zoliflodacin (PDB ID 8BP2) as well as a related compound QPT-1 (**6**, Figure 3, PDB ID 5CDM) [37,38]. Advances in DNA intercalating topoisomerase inhibitors including spiropyrimidinetriones have been reviewed elsewhere [39,40].

In addition to NBTIs and spiropyrimidinetriones, significant work has been produced studying inhibition of alternative binding sites of topoisomerases, including that of the second catalytic domain within DNA gyrase and topo IV, the ATPase domain found in GyrB/ParE, as well as a novel allosteric site identified in the vicinity of the GyrA active site. Through the targeting of distal sites within topoisomerases, it may be possible to reduce the current usage of FQs, thereby extending their lifespan and giving potential for combined-therapeutic treatments targeting DNA gyrase and topo IV. This review aims to summarise the research to date primarily focussing on non-intercalating inhibitors targeting alternative sites in bacterial topoisomerases.

## 2. Allosteric Inhibitors of GyrA

**Figure 4:**
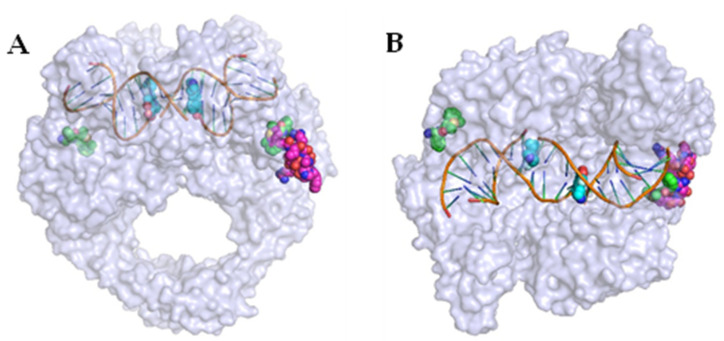
(**A**) Front view of superimposed DNA gyrase crystal structures highlighting the primary active site of ciprofloxacin (cyan spheres, PDB ID 2XCT [42]), and a novel allosteric site recently identified (green spheres, PDB ID 5NPP [41]). A further natural product, evybactin, was found to occupy the same allosteric site in *Mycobacterium tuberculosis* (magenta spheres, PDB ID 7UGW [43]). (**B**) Top view of the above-listed superimposed DNA gyrase crystal structures, rotated 90° forwards from (**A**).

**Figure 5:**
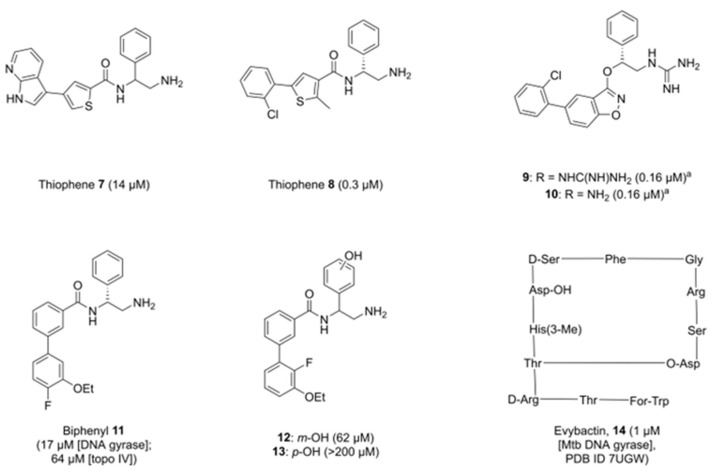
Inhibitors of bacterial DNA gyrase’s allosteric pocket, with IC_50_ values against *Escherichia coli* DNA gyrase in brackets unless stated otherwise. Thiophene compounds **7** and **8** were the basis for further developments into a fused ring system as well as a biphenyl system, whereas evybactin was identified as an antimycobacterial compound before the target was elucidated. ^a^A different assay was used to test the IC_50_ of **9** and **10** compared to all other compounds noted here [44], which utilised fluorescence spectroscopy in comparison to an agarose-gel-based assay used to test compounds **7**, **8**, **11**–**13**. As a result, they appear to be comparatively better, but are noted as being less potent than thiophene **8**, which was found to have an IC_50_ value of 0.04 μM when tested in the same assay conditions as **9** and **10**.

Researchers at GSK identified an allosteric site in DNA gyrase in 2017, providing a potential route to novel alternatives to FQs. A high-throughput screen by Chan et al. identified a thiophene-bearing compound, which was subsequently shown to bind to DNA gyrase in a previously unidentified pocket, dubbed the allosteric site, at the interface between GyrA and GyrB in the vicinity of the active site of the enzyme (Figure 4) [41]. Optimisation of the structure from **7** to **8** (Figure 5) improved activity across a range of bacterial species, including both Gram-positive bacteria (*S. aureus, Streptococcus pneumoniae*) and Gram-negative bacteria (*Acinetobacter baumannii, Pseudomonas aeruginosa, E. coli, Klebsiella pneumoniae*). Efflux of both compounds was proposed, with inhibition in efflux pump mutants of *E. coli*, *P. aeruginosa,* and *A. baumannii* improving efficacy by up to 64-fold.

Importantly, **8** was found be unaffected by mutations to key residues in GyrA, ParC, and ParE, with potent inhibition observed in FQ-resistant strains. However, one alternate mutation in GyrB-Glu^793^ (*E. coli* numbering, Glu^634^ in *S. aureus*) was identified which reduced inhibition caused by **8**. Further studies confirmed that the mechanism of action was different to that of FQs, as single- and double-strand DNA cleavage complexes were formed in equal quantities, as opposed to the entirely double-strand cleavage caused by the actions of ciprofloxacin. Although compound **8** showed promise due to its novel mode of action, this particular series was discontinued due to in vivo toxicity issues [41].

The series was altered to incorporate fused ring systems in place of the thiophene-amide motif, with the authors hypothesising that constraining the core would improve activity in biochemical assays. Moderate effects were observed in some analogues in the series [13]. Of note, benzisoxazole **9** (Figure 5) showed weak activity against *P. aeruginosa* and *K. pneumoniae*, whilst the primary amine analogue **10** (Figure 5) showed modest improvements against hERG and Na_v_1.5 channels when compared to **8**. However, the reduction in cytotoxic properties was not significant, with **10** still showing hERG and Na_v_1.5 IC_50_ values of 23 μM and 22 μM, respectively. Two crystal structures were generated of compound **9** in the allosteric site of topoisomerase with slight variations in the binding modes (PDB IDs 6QX1 and 6QX2). Of note, the chiral phenyl ring was found to adopt a rotated conformation within the pocket compared to parent structure **8**.

A further chemical series derived from thiophene **8** by Orritt et al. replaced the 5-arylthiophene with a biphenyl moiety, and explored changes to the chiral phenyl ring [45]. Although these compounds were not as potent as compound **8** on DNA gyrase (activity for lead compound **11** was 17 μM, cf. 0.3 μM for **8**), compound **11** (Figure 5) was found to have dual activity, targeting both DNA gyrase and topo IV (64 μM, cf. >540 μM for **8**). Current allosteric inhibitors of topoisomerases are designed around knowledge of the DNA gyrase crystal structures (PDB IDs 5NPK, 5NPP), but less is understood about the structure of the equivalent allosteric pocket in topo IV, other than a high degree of amino acid identity. Therefore, SAR data around allosteric inhibition of topo IV could prove beneficial in the development of dual-targeting topoisomerase inhibitors.

It was also observed that whilst the *R*-enantiomers of this series were more potent than the *S*- or racemic counterparts, enantiomeric *S*- and racemic compounds tested showed reasonable activity, with some activities of *S*-enantiomers in the same order of magnitude as their *R*-counterparts. Moreover, functionalising the chiral phenyl ring with a phenol moiety did not give any benefit in either the *meta*- or *para*-position (**12** and **13**, Figure 5). It was predicted that a phenol group would create an additional interaction with Arg^630^ through an intermediary water, but activity was found to drop, though **12** still retained micromolar activity [45].

Imai et al. utilised a different method to identify a novel natural product capable of inhibiting *Mycobacterium tuberculosis* (Mtb) DNA gyrase [43]. By screening extracts taken from bacterial strains symbiotic with a nematode host, the researchers were able to identify and characterise the depsipeptide evybactin (**14**, Figure 5), a cyclic peptide of 12 amino acids, which was shown to have good activity against Mtb (IC_50_ = 1 μM Mtb DNA gyrase, MIC = 0.0625 μg mL^−1^), but not eukaryotic cell lines. Subsequent analysis and X-ray crystallography showed evybactin to inhibit DNA synthesis by binding to Mtb DNA gyrase at a site overlapping with the allosteric pocket identified by Chan et al. (PDB ID 7UGW) and showed no cross-resistance with known FQ sites. Interestingly, evybactin was found to target Mtb specifically due to its transport into the cell. The transport protein BacA, capable of moving highly hydrophilic molecules across the outer membrane [46], was able to move evybactin into the cytoplasm. In cells lacking BacA or its homolog SbmA, antibacterial and antimycobacterial activities were weak. Even in other organisms bearing SbmA, such as *E. coli*, the presence of other efflux mechanisms such as TolC nullifies the activity of evybactin, making it specific to mycobacteria (MIC *E. coli* ATCC 25922 = 8 μg mL^−1^, MIC *E. coli* Δ*tolC* = 0.25 μg mL^−1^). Given the complex structure of evybactin, small molecules which can mimic its binding interactions would be highly desirable to combat Mtb.

## 3. Inhibitors of the ATPase Site of GyrB

**Figure 6:**
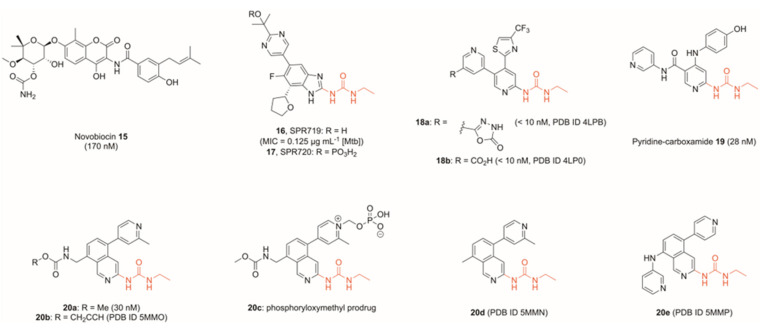
Examples of topoisomerase ATPase domain inhibitors with IC_50_ values against *E. coli* DNA gyrase unless stated otherwise. An ethyl urea moiety (red) was identified as an important motif capable of forming two key interactions with a conserved aspartate residue, and was subsequently used by multiple researchers in the development of novel topoisomerase inhibitors.

Inhibition of the ATPase activity of the GyrB subunit was identified as a potential route to new antibiotics as early as the 1950s. The discovery of the aminocoumarin group of natural product-derived antibiotics led to the approval of novobiocin (**15**, Figure 6), which reversibly inhibits DNA gyrase and, to a lesser extent, topo IV, through blocking of the ATPase site. This prevents the binding of ATP, metabolism and release of ADP, and the actions associated with this in the catalytic cycle. To date, novobiocin is the only aminocoumarin approved for clinical use, although it has now been withdrawn due to safer, more potent alternatives being developed, particularly later-generation penicillins. Interestingly, novobiocin has recently been studied for its anti-tumour effects as a result of its strong binding to the ATPase domain in DNA polymerase θ, with specific activity over other eukaryotic proteins bearing homologous ATPase domains including HSP90, suggesting a potential repurposing route for the drug [47].

Novobiocin’s validation as a topoisomerase inhibitor has, however, enabled the development of numerous subsequent GyrB/ParE inhibitors, many of which were based upon conserved motifs. The historic development of coumarins and other GyrB/ParE inhibitors has been reviewed by Bisacchi et al., which highlights the level of work which has gone into inhibiting this target [48]. 

Heterocycles bearing a *N*-ethylurea moiety were identified by Vertex in 2002 as a potent motif for binding to Asp^73^ in the adenine-binding region of DNA gyrase [49]. Gradual optimisation of the Vertex scaffold produced compound **16**, referred to as SPR719 (Figure 6), which displayed strong antibacterial properties (MIC_90_ = 0.032 μg mL^−1^ of *S. aureus*) [50], particularly against mycobacteria including Mtb (MIC_90_ = 0.125 μg mL^−1^ of extensively drug-resistant Mtb strain XDR 5) [51], though poor solubility limited its application. Development of prodrug **17** (SPR720, Figure 6), however, overcame these solubility issues, and a promising phase I trial showed minimal side-effects and good tolerability in healthy subjects, with the results indicating that **17** shows potential as an oral treatment of Mtb subject to further trials [52]. Compound **17** progressed to Phase II trials, and is currently being tested for treatment in mycobacterium avium complex pulmonary disease (NCT number NCT05496374).

Since this first application of the *N*-ethylurea motif, it has been used frequently in a range of gyrase ATPase inhibitors. A fragment-to-lead program by Basarab et al. incorporated this motif into a thiazole-bearing compound **18a** (Figure 6), which showed nanomolar activity against both Gram-positive and Gram-negative bacteria in a protein-based assay (IC_50_ <10 nM *S. aureus* and *E. coli* GyrB), though was only able to kill *E. coli* in an efflux pump mutant (MIC = 0.15 μg mL^−1^ of *E. coli* Δ*tolC*, MIC >64 μg mL^−1^ of *E. coli* wildtype) [53]. Significantly though, **18a** showed high potency against a range of *S. aureus* strains, including one methicillin- and quinolone-resistant (MIC = 0.06 μg mL^−1^).

Yule et al. further utilised the *N*-ethylurea motif to design and optimise a series of potent GyrB inhibitors. Using an in silico de novo design, the authors produced a pyridine-carboxamide (**19**, Figure 6), which showed nanomolar activity against GyrB (IC_50_ = 24 nM *S. aureus* GyrB and 28 nM *E. coli* GyrB in a protein-based assay) and ParE (IC_50_ = 86 nM *S. aureus* ParE and 940 nM *E. coli* ParE) [54]. In all cases listed above, bioengineered mutants confirmed GyrB/ParE activity, with GyrB mutation T173N (*S. aureus* numbering) frequently found to reduce potency across the described series. GyrA/ParC mutations did not compromise activity, confirming the target to be the GyrB/ParE subunit. Moreover, an assay monitoring the frequency of spontaneous mutation of **18a** in *S. aureus* found mutation T173N to spontaneously occur in two of the three experimental conditions [53].

In another fragment-based approach, Panchaud et al. varied the scaffold by replacing the key pyridine group of compounds **18a**, **18b,** and **19** with an isoquinoline, whilst maintaining the *N*-ethylurea moiety (**20a**, Figure 6) [55]. Although the project direction saw optimisation geared towards gyrase inhibition, topo IV was further inhibited significantly (**20a** IC_50_ *E. coli* DNA gyrase = 0.03 μM, IC_50_ *E. coli* topo IV = 8 μM). In both Basarab and Panchaud’s work, X-ray crystal structures were obtained of key compounds in the target active site. In the case of Basarab, compounds **18a** and **18b** were crystallised in the ParE subunit (PDB IDs 4LPB, 4LP0, Figure 6), whilst in that of Panchaud, compounds **20b**, **20d** and **20e** were visualised in the GyrB subunit, allowing for key structural data to be observed in topo IV and DNA gyrase, respectively (PDB IDs 5MMO, 5MMN, 5MMP, Figure 6). In both cases, key interactions were observed between the pyridyl nitrogen and a water molecule, whilst the *N*-ethylurea moiety formed two interactions with a conserved GyrB aspartate residue.

The solubility of many of these ATPase inhibitors was found to be a hurdle in the development of the series. Hit compound **18a** showed an aqueous solubility of approximately 9 μg mL^−1^, and whilst replacement of the oxadiazolone head with a carboxylic acid (**18b**) improved the solubility 40-fold, the solubility was still below 1 mg mL^−1^ [53]. Panchaud et al. similarly found that their hit compound **20a** had a solubility of 20 μg mL^−1^ in a phosphate buffer, but development of the compound into a prodrug through the introduction of a phosphoryl group improved the solubility to 12.7 mg mL^−1^ (**20c**, Figure 6) [55]. Although similar to the method of optimising SPR719, these two prodrugs were developed independently, suggesting that this could be a promising approach to improving the physicochemical properties of GyrB ATPase inhibitors.

**Figure 7:**
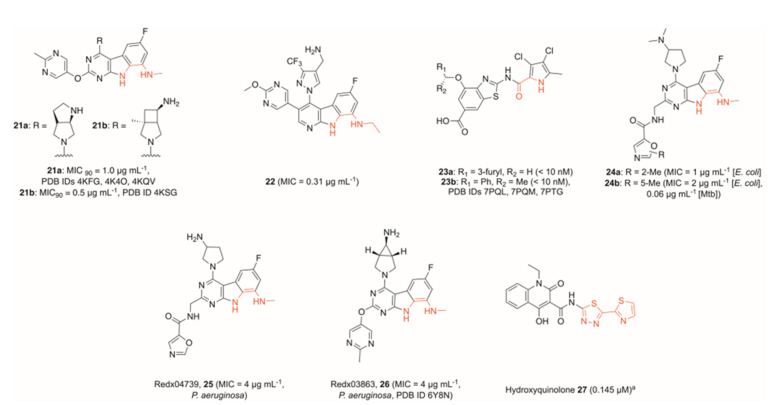
Development of bacterial topoisomerase ATPase inhibitors featuring novel core scaffolds, including tricyclic cores. Motifs binding to a conserved aspartate (red) branched away from the previously identified *N*-ethyl urea moiety. Values in brackets are IC_50_ values measured against *E. coli* DNA gyrase unless otherwise stated. Although no crystal structures were obtained for many of these compounds, conserved motifs were inferred due to comparisons with successfully crystallised analogues: **22** with PDB IDs 6M1S, 6M1J; **23** with PDB ID 6TTG, **24**–**26** with PDB ID 6Y8N. ^a^No crystal structure of **25** or similar analogues have been obtained, so the key binding atoms were not fully elucidated. As such, the entire thiadiazole-thiazole motif is highlighted to indicate that this region binds to the key aspartate residue, though the exact atoms remain undetermined.

Further developments moved away from the previously prominent *N*-ethylurea moiety, with other researchers looking towards tricyclic cores. A fragment-based drug discovery program by Trius Therapeutics produced two potent pyrimido-indoles bearing a methylamine functionality, capable of mimicking the interaction between the *N*-ethylurea motif previously utilised (**21a** and **21b**, Figure 7) [56]. These were found to be highly potent against Gram-positive bacteria, as well as additionally retaining good activity against a range of Gram-negative bacteria. This included strong potency against *S. aureus* (MIC_90_ **21a** = 0.06 μg mL^−1^, MIC_90_ **21b** = 0.008 μg mL^−1^) and *E. coli* (MIC_90_ **21a** = 1.0 μg mL^−1^, MIC_90_ **21b** = 0.5 μg mL^−1^). Compound **21a** was crystallised in the GyrB subunit of *E. coli* and *Enterococcus faecalis* (PDB IDs 4KFG, 4K4O) and the ParE subunit of *Francisella tularensis* (PDB ID 4KQV), whilst **21b** was crystallised in the GyrB subunit of *E. faecalis* (PDB ID 4KSG).

Hu et al. developed a *N-*ethylurea-containing scaffold into a pyrido-indole scaffold, which displayed dual activity against both GyrB and ParE [57]. This led to the generation of **22** (Figure 7), which had excellent potency against Gram-negative strains (MIC = 0.31 μg mL^−1^ of *E. coli*), and showed promise in a neutropenic mouse thigh infection model. A further effort to dual-target GyrB and ParE by Durcik et al. stemmed from a benzothiazole core identified previously [58,59]. The lead compound **23a** (Figure 7) showed excellent activity against both DNA gyrase and topo IV across a range of Gram-positive and Gram-negative bacteria (IC_50_ <10 nM *E. coli* and *S. aureus* DNA gyrase, IC_50_ = 54 nM *E. coli* topo IV and 84 nM *S. aureus* topo IV), whilst also showing an excellent cytotoxicity profile. Further development of this series yielded compound **23b** (Figure 7), which showed excellent activity against Gram-negative bacteria [60]. The lead compound was highly active against both DNA gyrase and topo IV in a range of Gram-negative bacteria and performed excellently against wildtype Gram-negatives in addition to efflux-deficient strains in killing assays (MIC = 1.0 μg mL^−1^ of *E. coli*, *K. pneumoniae,* and *P. aeruginosa* wildtypes, MIC = 0.5 μg mL^−1^ of *A. baumannii* wildtype). The authors note, however, that attempts to optimise physicochemical and ADME properties proved difficult, meaning properties such as solubility and plasma-free fraction were not suitable for in vivo testing. Crystal structures were obtained, though, for compound **23b** in the GyrB subunit of *A. baumannii* (PDB IDs 7PQL [racemate] and 7PQM [(*S*)-enantiomer]), as well as *P. aeruginosa* (PDB ID 7PTG [(*S*)-enantiomer]).

McGarry et al. developed a tricyclic GyrB inhibitor, which instead featured a pyrimido[4,5-b]indol-8-amine core [61], derived from previous work into GyrB inhibitors (PDB ID 4K4O) [62]. Mimicking the interactions between the urea moiety and GyrB-Asp^73^ of compounds **16**–**20**, the authors utilised the 8-methylamino group and *N*-indole functionalities of **24a** and **24b** (Figure 7) to create two hydrogen bonds, as well as creating the hydrogen bond between the previously identified water molecule and pyrimidyl moiety. In addition to identifying a novel GyrB inhibitor scaffold, the authors showed strong activity against Mtb (MIC **24b** = 0.06 μg mL^−1^), and improved activity within the series by extending into a previously unexplored pocket in the direction of Asp^106^. The SAR series showed that a carboxamide-oxazole side-chain was preferable, and although the addition of a methyl group at either free site on the oxazole improved efficacy, a molecular dynamic simulation suggested that **24a** displays fewer steric clashes than **24b**, and, hence, a 2-methyl moiety is preferential. 

However, subsequent work by Henderson et al. determined compound **25** (Redx04739, Figure 7) of the same series to be slightly less potent than a previously described analogue **26** (Redx03863, Figure 7) across a range of Gram-positive and Gram-negative bacterial strains. **26** showed notable activity against *P. aeruginosa*, with an MIC value of 4 μg mL^−1^ [63]. Although this value is higher than activities against other tested strains, *P. aeruginosa* employs a range of defence mechanisms, particularly multiple efflux pumps, which significantly reduces the impact of tested drugs. As such, reasonable activity against this bacterium is a marked improvement over several other topoisomerase inhibitors, including novobiocin and several tested oxazole-bearing tricycles [61]. Moreover, the authors successfully crystallised **26** and novobiocin in the *Mycobacterium thermoresistibile* GyrB ATPase sub-domain, as well as novobiocin in the *Mycobacterium smegmatis* GyrB ATPase sub-domain, allowing them to determine that the two compounds occupy a similar binding site with some overlap, but in general, the tricycle binds deeper into the pocket (PDB IDs 6Y8N, 6Y8L, 6Y8O, respectively). It also makes distinct interactions with GyrB compared to novobiocin, leading to differences in resistance patterns. For example, novobiocin forms a key bonding interaction with Arg^141^, but site-directed mutagenesis proved that interaction with this residue is not a requirement for **26** to maximise its potency.

**Figure 8:**
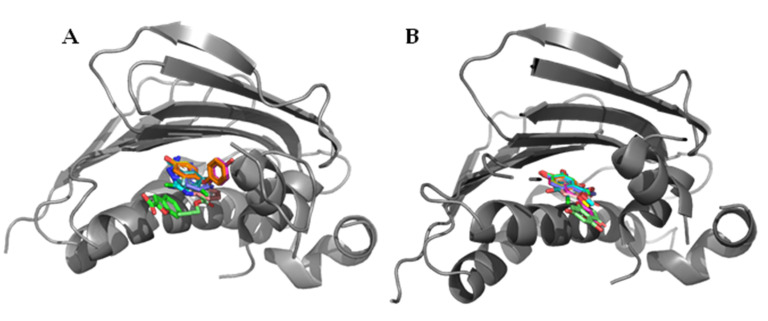
Superimposed co-crystal structures of fragments bound to the ATPase sub-domain of DNA gyrase identified by (**A**) Huang et al. and (**B**) Yu et al. [64,65]. A wider range of positions and functional groups were found in crystals produced by Huang et al., including one fragment (green) which filled a similar space to novobiocin. In comparison, the work by Yu et al. found one dominant binding mode, but multiple examples of phenols in this position suggest it may be a favourable binding mode for fragments of this class (PDB IDs (**A**): 5Z9N, 5Z9N, 5Z4H, 5Z9L, 5Z9P, 5Z9Q, 5Z9F, 5Z4O, 5Z9E, 5Z9B; PDB IDs (**B**): 7DOR, 7DPR, 7DPS, 7DQF, 7DQH, 7DQI, 7DQJ, 7DQL, 7DQM, 7DQS, 7DQU, 7DQW).

Fragment screens have been successfully deployed multiple times in the search for GyrB/ParE inhibitors, in part due to the presence of numerous hydrophilic and charged residues in the active site, as well as the availability of crystal structures of the GyrB/ParE subunits. Huang et al. employed a fragment screening approach to identify novel motifs capable of binding to the GyrB subunit, and novel druggable sites [64]. Through a combination of a thermal-shift assay and an ATPase-activity screen, 49 fragments were identified from a library of 486, of which 10 were successfully co-crystallised into the *E. coli* GyrB ATPase sub-domain, highlighting the high hit-rate a fragment-based approach can yield (Figure 8). 

Multiple binding modes were identified from the 10 crystallised fragments, with some showing micromolar activity and others showing no activity up to 1 mM. Some active compounds were found to overlap with novobiocin’s binding mode, whilst others bound deeper into the pocket. This research could provide a basis for a fragment merging approach in the future to explore the effect of combining these alternate binding sites to explore both the active and inactive fragments as potential sites within topoisomerases to boost activity.

Yu et al. adopted a similar approach to identify and co-crystallise 12 ATPase inhibiting fragments, of which the two most active compounds were found to have high micromolar activity (Figure 8) [65]. Phenols were a common motif in this screen, with all 12 successfully crystallised ATPase binders containing at least one. Whilst this does indicate that a phenol is a suitable fragment core for the binding site, the fragment screen led to limited modes of binding being identified, with eight of the fragments adopting a similar binding pose deep in the ATPase binding pocket.

Interestingly, Xue et al. utilised a different technique to identify a suitable fragment to build from. Instead of screening a fragment library, the group progressed from one single active molecule, identified through their previous work, and after predicting its interactions in the GyrB catalytic site, fragmented that to a 4-hydroxy-2-quinolone core [66,67]. Working from this lone fragment, the authors then performed a virtual screen on compounds bearing this motif and ultimately identified several compounds displaying sub-micromolar activity, including **27** (IC_50_ DNA gyrase = 0.145 μM, Figure 7). The hit compounds tested showed reasonable antibacterial activity against a range of *S. aureus* strains, though optimisation of solubility alongside other physicochemical properties is desirable. The redox potential of the scaffold may further warrant exploration into potential off-target effects.

## 4. Miscellaneous Topoisomerase Inhibitors

**Figure 9:**
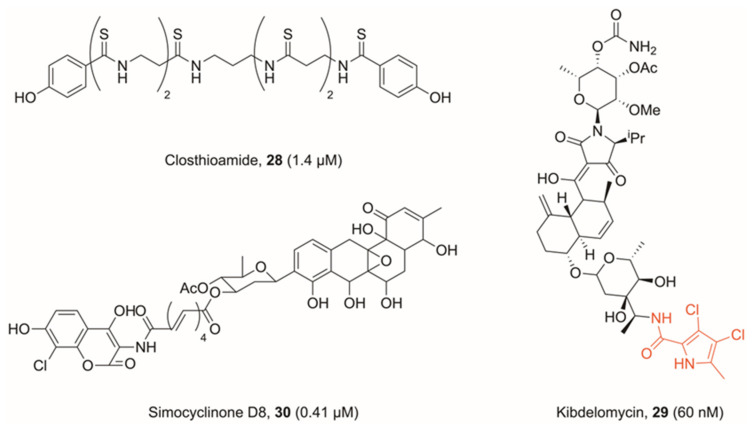
Bacterial topoisomerase inhibitors displaying unique or unusual modes of inhibition, with IC_50_ values against *E. coli* DNA gyrase in brackets. Closthioamide and kibdelomycin are ATPase inhibitors, though display different modes of inhibition to known ATPase inhibitor novobiocin. Simocyclinone D8 is further found to bind to a region of GyrB; hence, it displays a novel mechanism of action where it is capable of interacting with two regions of DNA gyrase simultaneously.

Several natural products have been previously identified, which branch away from the modes of inhibition outlined above. The first, a polythioamide produced by *Clostridium cellulolyticum* called closthioamide (**28**, Figure 9), was found by Chiriac et al. to be a strong DNA gyrase inhibitor (IC_50_ *E. coli* = 1.4 μM), with moderate potency against topo IV (IC_50_ *E. coli* = 113 μM) [68,69]. The authors’ findings show that closthioamide likely has a separate mode of inhibition to both FQs and novobiocin. However, their suggestion is that it is inhibiting the ATPase activity of gyrase, though more likely at a site distal to the novobiocin binding site. Comparisons were drawn with a further natural product kibdelomycin (**29**, Figure 9), derived from *Kibdelosporangium* sp. (MA7385), which also shows ATPase inhibitory activity, whilst acting with a different mode of inhibition to novobiocin [70]. Kibdelomycin occupies a similar region to novobiocin in the ATPase domain, but shows no cross-resistance. This is because of a unique binding mode, which does not utilise GyrB-Asp^89^ (*S. aureus* numbering) to form a bonding interaction, which is key to many other DNA gyrase ATPase inhibitors. The natural product displays a U-shaped conformation, which shares no common features to the way in which novobiocin binds to the same region, and hence its ATPase inhibition differs from that of novobiocin [71]. Kibdelomycin features an interesting dichloropyrrole motif, which was identified by researchers at AstraZeneca as a potent binder to the adenine pocket of GyrB/ParE, and subsequently benzothiazole **23a** and **23b**, highlighting its versatility across scaffolds [59,72]. Kibdelomycin displays strong inhibition against DNA gyrase in proteinogenic assays (IC_50_ = 60 nM *E. coli*), though this does not translate to potency in Gram-negative bacteria (MIC >64 μg mL^−1^ of *E. coli*) [70].

The natural product simocyclinone D8 (**30**, Figure 9), derived from *Streptomyces antibioticus* Tü 6040, has further been the subject of much research. This bifunctional molecule features both an aminocoumarin head and polyketide, and studies have found that it binds in two sites on DNA gyrase [73]. The primary site on the N-terminal domain of GyrA is occupied by the polyketide head, whilst the aminocoumarin head sits on the C-terminal domain of GyrB [74]. However, simocyclinone D8 is unable to competitively inhibit ATPase activity in GyrB, nor does it promote cleavage complex formation. Therefore, it has a unique mechanism of action with respect to both binding sites.

X-ray crystallography shows that the polyketide head does indeed occupy a space adjacent to ciprofloxacin, and hence likely intercalates DNA, but the site is nonetheless distinct. In fact, the dominant mechanism of action of simocyclinone D8 is to prevent cleavage complex formation, in stark contrast to FQs which allow formation and then stabilise the cleavage complex. Although simocyclinone D8 does also bind to a site on GyrB, this is the minor binding site, and whilst it cooperatively functions with binding on GyrA, the GyrB binding is calculated to be approximately 1000-fold weaker [75,76]. It further shows good activity against *E. coli* in in vitro testing (IC_50_ = 0.41 μM *E. coli* DNA gyrase) [77].

The natural products reported in this section have all been characterised as bacterial topoisomerase inhibitors via macromolecular biosynthesis and supercoiling DNA gel-based assays with ciprofloxacin and novobiocin as controls. To our knowledge, their potential off-target effects have not been characterised with respect to structural features such as Michael acceptors and redox-active aromatics. Therefore, follow-up characterisation is warranted to rule out chemical promiscuity or pan-assay interference before pursuing further as bacterial topoisomerase inhibitors [78].

## 5. Conclusions

Although improvement upon and development of new FQs will prove fruitful in the generation of new antibiotics, other approaches are required to prevent the antibiotic pipeline from drying up. Topoisomerases are a known target, which have been validated in both Gram-positive and Gram-negative bacteria, making them an excellent drug target. This review has summarised key research into topoisomerase inhibitors with different modes of action to FQs, particularly those which target a recently discovered allosteric site in the GyrA/ParC subunit, and GyrB/ParE inhibitors which bind to the ATPase site. Moreover, a discussion of compounds with novel modes of action highlighted that other DNA-intercalating compounds exist which do not proceed via the FQ mechanism of action, nor that of NBTIs. The research highlighted above shows that the scope for novel topoisomerase inhibitors is broad, and future research may produce new life-saving antibiotics, with some research mentioned already in clinical trials.

## Data Availability

Data sharing not applicable.
